# Current Research Trends and Hotspots in Radiotherapy Combined with Nanomaterials for Cancer Treatment: A Bibliometric and Visualization Analysis

**DOI:** 10.3390/nano15151205

**Published:** 2025-08-06

**Authors:** Muyasha Abulimiti, Shiqin Dai, Ebara Mitsuhiro, Yu Sugawara, Yinuo Li, Hideyuki Sakurai, Yoshitaka Matsumoto

**Affiliations:** 1 Department of Radiation Oncology, Graduate School of Comprehensive Human Sciences, University of Tsukuba, Tsukuba 305-8575, Japan; drmuyasha@126.com (M.A.); yu-suga0315@outlook.com (Y.S.); 2 Research Center for Functional Materials, National Institute for Materials Science (NIMS), Tsukuba 305-0044, Japan; dsq980822@163.com (S.D.); ebara.mitsuhiro@nims.go.jp (E.M.); 3 Graduate School of Pure and Applied Sciences, University of Tsukuba, Tsukuba 305-8575, Japan; 4 Department of Materials Science and Technology, Tokyo University of Science, Tokyo 125-8585, Japan; 5 Proton Medical Research Center, University of Tsukuba Hospital, Tsukuba 305-8576, Japan; lyn19960714@hotmail.com (Y.L.); hsakurai@pmrc.tsukuba.ac.jp (H.S.); 6 Department of Radiation Oncology, Clinical Medicine, Institute of Medicine, University of Tsukuba, Tsukuba 305-8575, Japan; 7 Department of Radiobiology, Biomedical Science, Institute of Medicine, University of Tsukuba, Tsukuba 305-8575, Japan

**Keywords:** radiotherapy, nanomaterials, nanobiomaterials, cancer, drug delivery

## Abstract

This study investigated the evolving trends, current research hotspots, and future directions of radiotherapy combined with nanobiomaterials through a bibliometric analysis. Publications related to nanobiomaterials used in radiotherapy between 2004 and 2024 were retrieved from the Web of Science Core Collection database and analyzed using VOSviewer, R, and CiteSpace. China emerged as the leading contributor, accounting for 1051 publications (50.41%), followed by the USA. Liu Zhuang is the most productive author in this field. American Chemical Society (ACS) Nano published the most influential articles and accumulated the highest number of citations. Advanced Targeted Therapies in Cancer: Drug Nanocarriers, the Future of Chemotherapy was the most cited, with 1255 citations. Citation bursts have revealed emerging research trends in targeted delivery, cellular studies, co-delivery strategies, immunogenic cell death, polymeric nanoparticles, tumor research, and drug delivery systems, indicating potential avenues for future research. Over the past two decades, nanomaterials for radiotherapy have gained substantial attention. Key areas of focus include enhancing the efficacy of radiotherapy, achieving targeted drug delivery, minimizing adverse effects, and integrating nanomaterials with other therapeutic modalities. Future investigations are expected to improve the precision of radiotherapy, augment radiation effects, and optimize the tumor microenvironment.

## 1. Introduction

Nanomaterials have garnered significant attention owing to their potential for precise cancer diagnosis and treatment. These materials are typically characterized by particle diameters ranging from 1 to 100 nm [1]. Nanomaterials have been used in clinical settings for approximately 30 years. A notable example is doxorubicin liposome injection (Doxil), approved by the Food and Drug Administration (FDA) in 1995 [2,3]. This formulation encapsulates the chemotherapeutic drug doxorubicin in liposomes, which are nanosized lipid particles. This method improves drug accumulation at tumor sites while minimizing side effects in healthy organs. Additionally, nanomaterials are utilized for the early diagnosis and precise localization of tumors or diseased tissues, overcoming the limitations of conventional clinical detection and imaging methods.

Since the 2000s, gold nanoparticles (GNPs) have been used in radiotherapy. GNPs are recognized for enhancing X-ray absorption, enabling them to concentrate radiation energy specifically in tumor areas [4]. This targeted approach enhances radiation-induced cytotoxicity while minimizing adverse effects on surrounding healthy tissues. In addition to GNPs, the integration of other nanomaterials in radiotherapy and chemotherapy has yielded promising results. Nanomaterials can modify the tumor microenvironment and act synergistically with targeted immunotherapies. However, the outcomes of these approaches have been inconsistent. Despite these challenges, the use of nanomaterials in radiotherapy has shown significant promise. However, numerous challenges must be addressed before their widespread application in clinical settings. This article reviews the progress and emerging trends in the use of nanomaterials in radiotherapy over the past 20 years.

Bibliometric analysis is a statistical approach that uses publication data and the key literature to evaluate research developments [5]. This method provides an objective and comprehensive understanding of a research domain by systematically collecting relevant studies and applying statistical techniques. Unlike conventional reviews that are influenced by individual perspectives, bibliometric analyses offer quantitative and qualitative assessments. Although this method has not yet been applied to radiotherapy studies involving nanofiber technology, our study seeks to address this gap. This study aims to provide a comprehensive perspective, aiding researchers in understanding the historical evolution, current advancements, and future directions in this area.

## 2. Materials and Methods

### 2.1. Data Acquisition

**Figure 1:**
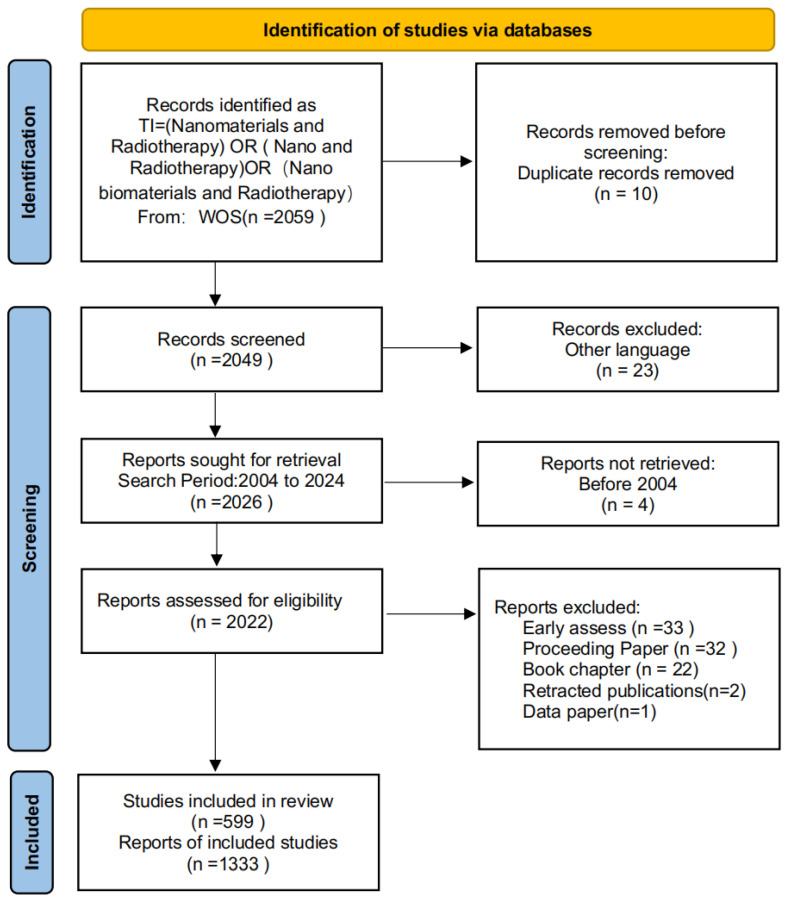
PRISMA workflow.

We used the Web of Science Core Collection (WoSCC) as our primary data source to ensure the reliability of the selected literature and maintain appropriate reference formatting. The search strategy employed the following query: TS = (nanomaterials AND radiotherapy) AND LA = (English) AND DT = (Article OR Review). The search encompassed English language articles and reviews published between 2004 and 2024. To maximize data retrieval, we incorporated multiple keyword variations, including “nanomaterials,” “nanobiomaterials,” and “NANO.” Following the PRISMA flowchart, a systematic selection process was conducted, and the final dataset was exported in a plaintext format. The study’s detailed PRISMA workflow is illustrated in Figure 1. To ensure transparency, the study protocol was registered in the Open Science Framework (OSF). The full registration record can be accessed via DOI: https://doi.org/10.17605/OSF.IO/6DQHA (accessed on 9 July 2025).

### 2.2. Bibliometrics and Visualization Analysis

Biometric analyses were performed using VOSviewer (version 1.6.20, Leiden University, The Netherlands), R (version 4.4.1), and CiteSpace (version 6.3.1, Drexel University, United States). VOSviewer was used to examine the statistical attributes of countries, institutions, journals, authors, and keywords, whereas CiteSpace was used to detect keywords and citation bursts. Data visualization was performed using specialized bibliometric software, including the R package, tailored for this purpose [6]. The dataset was retrieved from WoSCC via the “Export Records to File” function, selecting “Full Record and Cited References” as the export format, and saved as plain text or tabdelimited files for further processing. Key bibliographic details, including annual publication and citation counts, countries, institutions, authors, journals, funding sources, research areas, keywords, and references, were extracted and organized in a Microsoft Excel spreadsheet. Journal impact factors (IFs) and quartile rankings were obtained from the 2020 Journal Citation Reports (JCRs). The compound annual growth rate (CAGR) was calculated to describe the average annual increase in publication output during the study period:

$$\mathrm{CAGR}={\left(\frac{V_{f}}{V_{i}}\right)}^{\frac{1}{n}}-1$$

where $V_{f}$ is the final value, $V_{i}$ is the initial value, and *n* is the number of years between the two time points.

The H-index, which balances research productivity and impact, is defined as the number of papers (h) cited at least h times. For instance, an H-index of 20 indicates that 20 publications have received a minimum of 20 citations. Qualitative and quantitative analyses were performed using VOSviewer, CiteSpace, and an online bibliometric analysis platform (https://bibliometric.com (accessed on 5 February 2025)).

## 3. Results

### 3.1. Publication Characteristics

**Figure 2:**
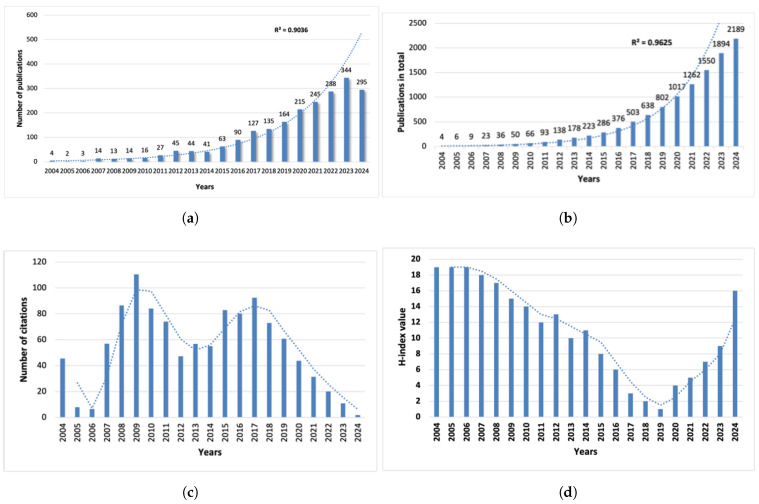
(**a**) Annual publication trends in the past 20 years. (**b**) Total number of publications over time. (**c**) Number of citations each year. (**d**) H-index each year.

A total of 2022 publications related to nanomaterials and radiotherapy were retrieved from the WoSCC (Table 1). The annual publication trends between 2004 and 2024 are shown in Figure 2a. From 2004 to 2011, the number of publications gradually increased, with annual increments not exceeding 10 articles. However, from 2012 onward, the number of publications has increased significantly, peaking at 344 articles by 2023. Although a slight decrease was observed in 2024, the number of articles remained high at 295. The R^2^ value was 0.9036, indicating that the growth trend was well described by the fitted curve (Figure 2a). This decrease may be partially due to the timing of data retrieval, which occurred in January 2025 and might not reflect the full indexing of the 2024 publications. Additionally, a potential shift in research interest cannot be ruled out, and continuous observations in the coming years are required to confirm whether this represents a short-term fluctuation or a broader trend. The number of published articles steadily increased from 2004 to 2024 (Figure 2b), corresponding to a CAGR of approximately 23.6%, indicating steady and substantial growth. From 2004 to 2024, the annual total citations were the highest in 2009 (Figure 2c), and the annual H-index remained above 15 and subsequently decreased to approximately 1 by 2019. By 2024, it was close to 16 (Figure 2d).

### 3.2. Countries/Regions and Institutions

**Figure 3:**
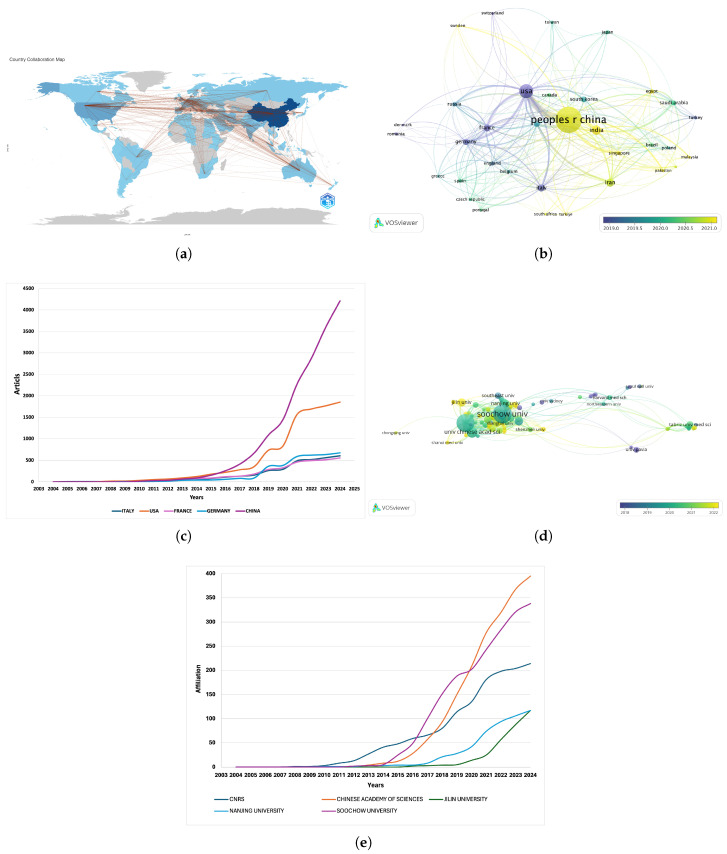
(**a**) Country collaboration map. (**b**) Collaboration network of 84 countries/regions with more than 10 publications. (**c**) Annual publication trends of top countries. (**d**) Institutional collaboration network. (**e**) Annual publication trends of top institutions.

A total of 84 countries have published studies on radiotherapy. The country collaboration map validated that this area of research was truly global, with connections spanning all continents (Figure 3a). Among these, 34 countries published more than 10 articles (Figure 3b), and the lines between the nodes represent the collaborative relationships between countries. Thicker and more numerous lines indicate stronger or more frequent connections. The USA is closely linked to European countries such as Germany and France, whereas China has stronger connections with countries in Asia (e.g., India, the Republic of Korea) and the Middle East. The color gradient (from blue to yellow) in the map represents the timeline of the research activity. Asian countries have demonstrated growing interest in this field over the years. The link strengths (LSs) between nodes reflect the degree of cooperation between countries, and the total link strength (TLS) is the sum of the LSs for a specific node. China produced the most publications (1051) and had the highest number of total citations (47,286). The USA had the highest TLS (202), followed by China with a TLS of 172) (Table 2). The trend observed between 2004 and 2024 indicated that significant international attention to this topic began after 2014 (Figure 3c). Notably, China has experienced the most substantial growth, with a sharp increase beginning in 2015 and continuing to increase steeply beyond 2020. In addition, the USA has shown notable growth, albeit at a slower rate than China. By contrast, Italy, France, and Germany have relatively steady but much slower growth rates.

A total of 2386 institutions have contributed to research on nanofibers and radiotherapy. Figure 3d shows the 89 institutions with more than ten publications. The Chinese Academy of Sciences exhibited the most cooperative relationship (TLS = 105). The top ten most productive institutions are listed in Table 3. Soochow University was the most productive (155) and had the highest total citations (14,234), followed by the Chinese Academy of Sciences (142) and the University of the Chinese Academy of Sciences (65). Over the past 20 years, the Chinese Academy of Sciences and Soochow University have experienced a sharp increase in both their numbers of publications and citations.

### 3.3. Authors

**Figure 4:**
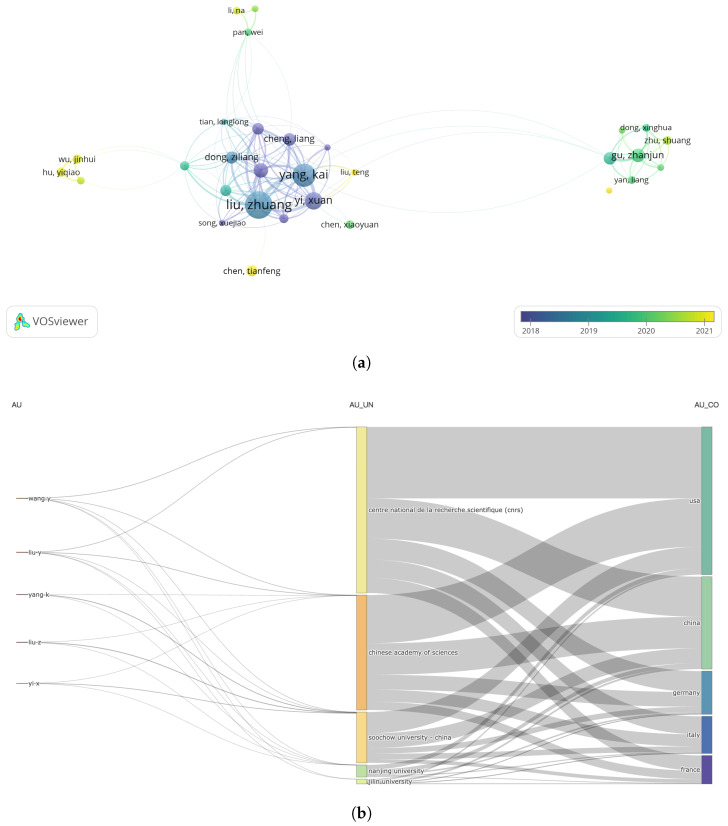
(**a**) Overlay network analysis of 45 researchers with at least 10 publications. (**b**) Sankey diagram of the relationships among the five most influential authors, institutions, and countries.

A total of 11,144 authors contributed to research on nanomaterials and radiotherapy. Table 4 lists the ten most productive researchers in this area. Liu and Zhuang exhibited the highest publication count (77), followed by Yang and Kai (61) and Yi and Xuan (41). In terms of academic impact, Liu and Zhuang ranked first, with 10,861 total citations and a TLS of 197. Figure 4a presents an overlay network analysis of 45 researchers who published at least 10 articles. Figure 4b illustrates the relationships among the top five authors, their affiliated institutions, and the countries contributing to nanomaterial and radiotherapy research between 2004 and 2024 using a Sankey diagram [7]. Chinese institutions play a dominant role in this field, demonstrating the highest level of research connectivity.

### 3.4. Journals

**Figure 5:**
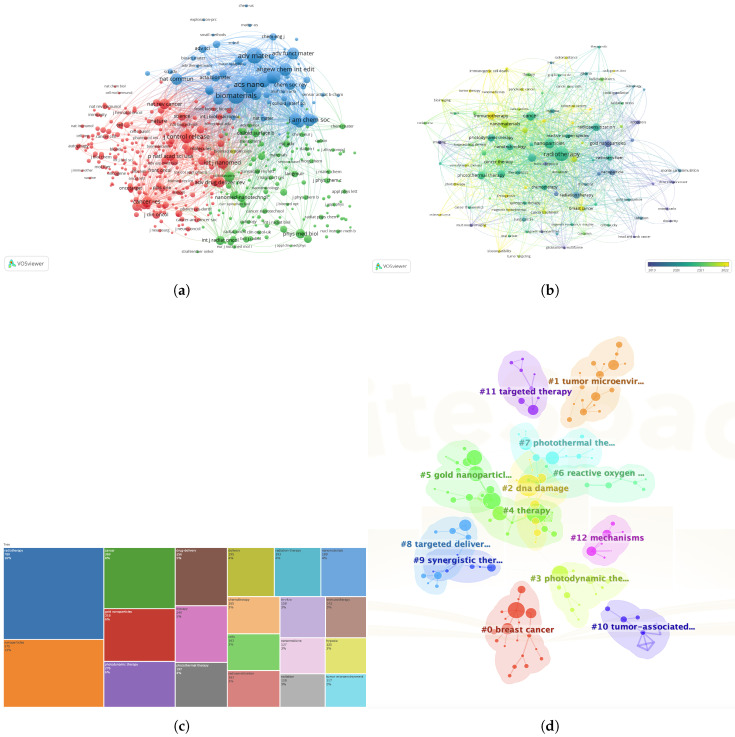
(**a**) Journals that have published more than 50 articles. (**b**) The minimized 20-time occurrence keywords. (**c**) The top common keywords. (**d**) Research topic clustering map.

A total of 9758 journals published articles on nanomaterials and radiotherapy. Among these, 461 journals published more than 50 articles on this topic. Network visualization presents the interconnections between journals and research topics, and different colors represent clusters of research areas. Nanomaterials, biomaterials, and drug delivery are the major research trends that form closely linked academic communities (Figure 5a). Table 5 presents the top ten journals, detailing their publication counts, total citations, average citations per publication, IF, and JCR quartile rankings. All the journals listed are JCR Q1 journals. ACS Nano produced the most publications (128) and the highest total number of citations (9255), followed by Advanced Materials, with the highest average number of citations (173) and the best IF score (IF = 32). Nano Letters and Advanced Functional Materials had relatively fewer publications but a high number average citations per paper, highlighting their significant impact.

### 3.5. Keywords

A total of 7836 keywords were identified. Of these, 162 have appeared at least 20 times. The 10 keywords used most frequently were “radiotherapy”, “nanoparticles”, “cancer”, “gold nanoparticles”, “photodynamic therapy”,“drug delivery”, “therapy”, “photothermal therapy”, “delivery” and “chemotherapy” (Figure 5b,c). The keywords displayed in dark blue represent an average publication year of 2019 or earlier, whereas those displayed in bright yellow represent 2022 or later. A research topic clustering map was created to explore the latest research trends. As shown in Figure 5d, 12 major clusters were identified, each representing a specific research domain. Closely related topics were placed near each other. Notable research themes include “breast cancer”, “targeted therapy”, “tumor associated”, and “photodynamic therapy”. Additionally, key topics such as “gold nanoparticles”, “reactive oxygen”, “DNA damage” and “targeted delivery” were also highlighted.

### 3.6. Citation Analysis

**Figure 6:**
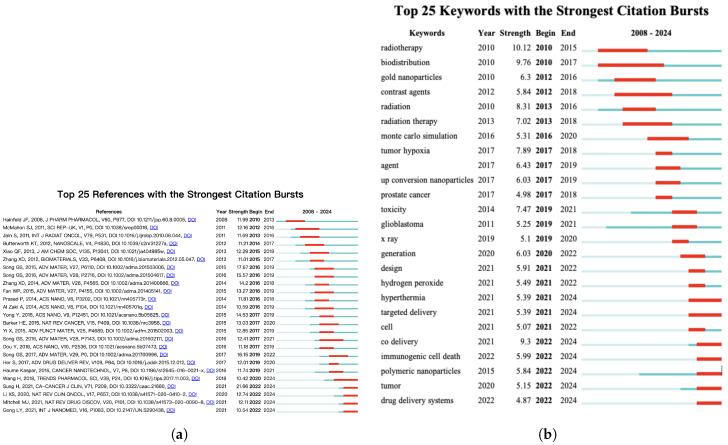
(**a**) Top 25 influential references with the strongest citation bursts from 2008 to 2024. (**b**) Top 25 impactful keywords with the strongest citation bursts from 2008 to 2024.

Citation analysis is an essential tool for assessing the impact of publications within a specific research field, with citation count serving as a key indicator of the impact of an article. Among the 75 most cited publications, 48 were review articles and 27 were original research articles. After filtering, 64 highly cited articles, including 45 reviews and 19 research articles, were selected for the analysis. The most cited publication among these was a review article titled “Chemical Design and Synthesis of Functionalized Probes for Imaging and Treating Tumor Hypoxia,” authored by Liu and published in Chemical Reviews in 2017. This review systematically summarizes the design principles and synthetic approaches for functional probes that target hypoxic tumor regions. The article has played a significant role in shaping research on hypoxia-responsive nanomaterials for cancer imaging and therapy, thereby advancing targeted treatment strategies. This article has received 607 citations. The 64 most cited publications are primarily distributed across the following research areas: 16 in Materials Science and Nanotechnology, 12 in Biomedical and Pharmaceutical Sciences, 6 in Chemistry and Interdisciplinary Sciences, and 8 in Cancer and Medical Research. Details are provided in the Supplementary Table S1. The most recent and strongest citation burst was observed for a publication by Sung (2021) with a burst strength of 21.66, occurring between 2019 and 2021 (Figure 6a). This study serves as a guide for researchers to align their work with urgent global health challenges. This strong citation burst reflects its widespread use, justifying the significance and urgency of cancer-related research, including nanomaterial-enhanced radiotherapy. The top keyword in the top 25 keywords with the strongest citation bursts (Figure 6b) was radiotherapy (2010), which had the highest citation burst strength (10.12) from 2010 to 2015. In the early stage (2008–2015), the research focused mainly on traditional radiotherapy techniques, biodistribution, and imaging-related terms such as “contrast agents” and “gold nanoparticles”. During 2015–2020, the attention turned toward characteristics of the tumor microenvironment, particularly “tumor hypoxia” and “toxicity”, as well as a specific type of cancer, “glioblastoma”. However, in recent years (2021–2024), emerging keywords such as “immunogenic cell death”, “co-administration”, “hyperthermia”, and “polymeric nanoparticles” suggest growing interest in multimodal treatment strategies, immune activation mechanisms, and the design of smart nanocarriers. This transition reflects the increasing emphasis on improving therapeutic precision, overcoming drug resistance, and integrating synergistic therapeutic modalities.

## 4. Discussion

### 4.1. Contributions by Countries, Institutions, Authors, and Journals

This review is the first comprehensive bibliometric analysis of nanomaterials in the context of radiotherapy. These findings indicate a consistent increase in scientific output over the past 20 years, with the highest number of publications occurring in 2023. Citation counts and the H-index exhibited varying growth trends, with notable peaks in citations around 2009 and 2017, and the H-index reached a significant low in 2019. Additionally, the analysis indicated that China has produced the most publications in this field, whereas the United States has the highest TLS, indicating its dominant influence and frequent citations within this research field. Nanomaterials combined with radiotherapy span a broad interdisciplinary topic encompassing multiple disciplines, including materials science, medicine, and chemistry. Liu Zhuang of Soochow University, China, has been identified as the most influential scholar in this field, and Soochow University is recognized as the leading institution. ACS Nano has published the highest number of articles on this topic. Advanced Materials, although second in publication volume, had the highest impact factor among the relevant journals.

### 4.2. Research Hotspots and Frontiers

Nanomaterials, which exist in the size range between the atomic and macroscopic levels, exhibit unique properties such as a high specific surface area, high reactivity, high strength, low thermal resistance, high specific heat capacity, high diffusivity, and special magnetic and optical properties [1]. These characteristics render nanoparticles exceptionally versatile for biomedical applications.

Based on the 20 most cited review articles and the most frequently occurring keywords over the past 20 years, research hotspots in this field can be broadly categorized in two primary directions. The first direction, nanomaterials used as radiosensitizers, includes photothermal therapy, photodynamic therapy, radiosensitizing materials, artificial intelligence, sonodynamic therapy, and catalytic oxygenation therapy. The second direction pertains to the use of nanomaterials in comprehensive cancer therapies, including targeted therapy, drug delivery, tumor microenvironment modulation, immunotherapy, and immune enhancement. Although we categorized research hotspots into two main domains, nanomaterials as radiosensitizers and nanomaterials for comprehensive cancer therapy, it is important to note that these categories are not mutually exclusive. For example, the use of nanomaterials in immunotherapy can enhance radiosensitivity and contribute to a comprehensive cancer treatment. Therefore, this classification provides a practical framework for understanding the literature, and the boundaries between these categories are inherently fluid and reflect the interdisciplinary and integrative nature of this evolving research field. Below, we provide a detailed overview of the research progress in these two areas.

#### 4.2.1. Nanomaterials as Radiosensitizers

Radiotherapy has gained traction in clinical settings to eliminate tumors through the application of localized ionizing radiation beams. However, the efficacy of radiotherapy is typically limited by tumor hypoxia-associated radiation resistance [8]. Additionally, radiotherapy has limited efficacy in controlling tumor metastasis, which is the primary cause of cancer-related mortality. Researchers have extensively investigated radiosensitization strategies to address relatively low radiation therapy efficiency. Radiosensitization refers to the process of augmenting the efficacy of radiation therapy by increasing the radiosensitivity of cancer cells [9]. This concept is central to oncology, as it has the potential to significantly improve patient outcomes. The mechanisms underlying radiosensitization are multifaceted and can be categorized at the molecular, cellular, and microenvironmental levels, all of which play crucial roles in enhancing the effectiveness of radiotherapy.

At the molecular level, tumor cells typically rely on DNA repair pathways to mitigate radiation-induced damage [10]. Radiosensitization can be achieved by inhibiting DNA repair pathways, particularly single- and double-strand breaks. Inhibition of key DNA repair proteins such as poly (ADP-ribose) polymerase (PARP), ataxia-telangiectasia mutated (ATM), and ATM- and Rad3-related (ATR) proteins can prevent the repair of radiation-induced damage, thereby enhancing radiosensitization [11]. At the cellular level, the primary objective of radiosensitization is to modify cellular response mechanisms to radiation-induced damage [12,13,14]. For example, the cell cycle influences cellular radiosensitivity, with cells in the G2/M phase exhibiting greater susceptibility to radiation-induced damage [15]. Strategies such as synchronizing tumor cells in the G2/M phase using drugs [16,17,18] or inhibiting cell cycle checkpoints using inhibitors can significantly improve sensitivity to radiotherapy [19,20]. Furthermore, the tumor microenvironment (TME) complicates the response to radiation. Hypoxia, an abnormal vasculature, and the presence of immune cells contribute to radiation resistance. Hypoxic regions are common in most solid tumors, exhibit radioresistant biological effects, and represent the most aggressive fraction of a tumor [21]. Several preclinical studies, both in vitro and in vivo, have indicated that decreasing oxygen concentrations induces resistance to radiation [22]. The TME enhances oxygen availability via strategies such as hypoxia-activated prodrugs or antiangiogenic therapies, thereby improving the tumor response to radiation therapy [23,24]. In addition, the immune microenvironment enhances radiosensitivity. Radiation therapy has the potential to induce immune responses that contribute to the eradication of tumor cells. By targeting DNA repair, cell cycle regulation, immune responses, signaling pathways, and the tumor microenvironment, researchers can mitigate radiotherapy resistance and improve the efficacy of radiotherapy.

Metallic radioactive nanoparticles (MRNPs) are a promising class of nanomaterials designed to enhance the efficacy of radiotherapy by improving radiation absorption, tumor targeting, and theragnostic capabilities [25]. These nanoparticles, typically composed of high-atomic-number metals, such as gold, silver, and platinum, are known for their ability to generate heat and produce reactive oxygen species. They enhance radiation absorption and produce secondary electrons that damage cancerous cells. By integrating radionuclides, such as Iodine-131, Yttrium-90, and Lutetium-177, MRNPs deliver targeted radiation directly to tumors, thereby minimizing damage to the surrounding healthy tissues [26]. Their high surface area and tunability enable the efficient loading of therapeutic agents, and functionalization with tumor-targeting ligands improves their specificity [27]. In addition to MRNPs, inorganic-based nanocarriers, including carbon nanotubes (CNTs), graphene oxide (GO), and fullerenes, have gained traction as radiosensitizers in cancer radiotherapy owing to their unique properties such as their high surface area, biocompatibility, and strong interaction with radiation. CNTs have been extensively studied because of their ability to enhance radiation therapy. Their high surface area and ability to absorb and scatter radiation result in the generation of secondary electrons, which enhance DNA damage in tumor cells [28]. Similarly, GO, which exhibits a two dimensional structure, has shown promise in radiotherapy by improving the delivery of radioactive isotopes, such as Iodine-131 or Yt-trium-90, and serving as a platform for the co-delivery of chemotherapeutic agents [29]. Fullerenes are another class of carbon-based nanoparticles that have been explored for their potential in radionuclide therapy owing to their ability to encapsulate radioactive agents and enhance their therapeutic effects. Carbon-based nanoparticles can be functionalized with targeting ligands to improve tumor specificity and reduce off-target toxicity [30]. Despite their potential, challenges such as biodegradation and immune system interactions remain, necessitating further investigations to ensure the safe and effective clinical application of CNTs in radiotherapy.

#### 4.2.2. Nanomaterials for Comprehensive Cancer Therapy

Tumor tissues are characterized by abnormal vascular proliferation, including high vascular density, poor vascular wall integrity, wide interstitial spaces, and slow lymphatic drainage. These features enable nanoparticles of specific sizes to penetrate and accumulate in tumor tissues, thereby achieving efficient and accurate enrichment at the tumor site. This phenomenon is known as the “enhanced permeability and retention (EPR)” effect, which is a form of passive targeting [31].This effect arises from the unique characteristics of the tumor vasculature. Unlike normal tissues, tumors often exhibit chaotic, highly permeable blood vessels with large fenestrations (ranging from 100 to 800 nm) that allow nanoparticles to extravasate more easily. In addition, tumors typically lack functional lymphatic drainage, which prevents the efficient removal of extravasated materials. These two factors, enhanced vascular permeability and impaired lymphatic clearance, together create a microenvironment that facilitates the passive accumulation and prolonged retention of nanoparticles at the tumor site. By attaching ligands that bind specifically to tumor markers (e.g., tumor-associated antigens or receptors), nanoparticles are directed to specific tumor sites, thereby facilitating drug accumulation within tumor tissues and reducing their distribution to nontumor areas. This approach converts passive targeting into active targeting. The common ligands include antibodies, small molecules, and aptamers. Polymeric and liposomal nanoparticles have also been widely studied for their potential to enhance cancer radiotherapy via improved drug delivery, circulation time-targeted therapy, and reduced systemic toxicity. Polymeric nanoparticles, typically composed of biocompatible polymers such as poly(lactic-co-glycolic) acid (PLGA) and polyethylene glycol (PEG), provide excellent stability, controlled release, and the ability to encapsulate both chemotherapeutic agents and radioactive isotopes [32]. These nanoparticles can be functionalized with targeting ligands to enhance tumor specificity and improve the therapeutic index. Liposomal nanoparticles, which are composed of lipid bilayers that encapsulate drugs or radionuclides, offer similar advantages by increasing the solubility of hydrophobic agents and providing a platform for multifunctional therapy. Liposomes can be loaded with radionuclides for targeted radiotherapy while facilitating imaging techniques such as magnetic resonance imaging (MRI) and positron emission tomography [33]. Both polymeric and liposomal nanoparticles have been designed to overcome challenges such as rapid clearance and nonspecific distribution by utilizing surface modifications such as PEGylation to extend the circulation time and enhance tumor accumulation [34]. Among the nanocarriers, liposomes and albumin-bound paclitaxel formulations are already applied in clinical settings [2]. Nanocarriers based on PLGA have been approved for specific clinical applications, such as lipid nanoparticles for vaccine delivery, including those used in COVID-19 vaccines [35]. Additionally, iron-based nanomaterials, such as superparamagnetic iron oxide nanoparticles and ferromoxytol, have been approved for use as contrast agents in MRI [36].

Furthermore, silica-based nanoparticles (SiNPs) have garnered significant attention in cancer radiotherapy owing to their unique physicochemical properties, including their high surface area, tunable porosity, and excellent biocompatibility [37]. They provide stable and biocompatible platforms for encapsulating radionuclides, thereby ensuring a controlled release and prolonged therapeutic effect [38]. The large surface area of SiNPs enables the efficient loading of radionuclides and chemotherapeutic agents, facilitating the controlled release and prolonged therapeutic effects at the tumor site [39]. Despite their promising potential, challenges related to the biodegradation and clearance of silica nanoparticles are being actively addressed through the development of biodegradable silica-based systems that aim to enhance their safety and efficacy for clinical applications [40]. Nanocarriers based on biological materials such as albumin nanoparticles are widely employed for long-term drug delivery and enhanced drug stability. Stimuli-responsive nanocarriers, which represent another promising class of materials, release drugs in response to specific stimuli, such as pH, temperature, light, the magnetic field, or sound, thereby enabling more precise and efficient drug delivery. Nanomaterials have also been explored for their use in immunotherapy. For instance, the interaction between CD47 and Signal Regulatory Protein (SIRP) is crucial for regulating the homeostasis of the immune system by acting as a “don’t eat me” signal that inhibits macrophage-mediated phagocytosis. Polymer-based nanoparticles have been used to display and block CD47 expression. Nanoparticles coated with CD47 can interact with SIRPα on macrophages to avoid clearance, while those designed to block CD47–SIRPα interactions can promote the phagocytosis of tumor cells by inhibiting this signaling pathway [41]. Beyond these applications, nanomaterials can modulate the tumor microenvironment and improve immune cell activity against tumors [42]. Researchers have used nanomaterials that encapsulate Stimulator of Interferon Gene (STING) agonists to improve their bioavailability and tumor penetration, which is crucial for effective immune modulation [43]. Nanomaterials used to improve the sensitivity and specificity of detecting Circulating Tumor Cells (CTCs) have emerged as a prominent research focus in immunotherapy and demonstrate significant potential [44]. Aljabali et al. developed various strategies using nanomaterials, including liposomes, polymers, and inorganic nanoparticles, highlighting the fact that nanomaterials can be designed for precise targeted delivery and to modulate the immune system for improved immunomodulatory efficacy [45]. The classifications and applications of nanomaterials for cancer treatment are summarized in Table 6.

### 4.3. Future Research Trends

As shown by the evaluation of bibliometric keywords, research interests have shifted from single nanomaterials and technology to interdisciplinary applications such as immunogenic cell death, co-delivery, and hyperthermia, reflecting a growing interest in multimodal treatment strategies, immune activation mechanisms, and the design of smart nanocarriers. Therefore, it is reasonable to anticipate that radiotherapy nanotechnology will focus on intelligent multifunctional systems that enhance treatment efficacy while minimizing side effects. A key area of focus is immune modulation, in which nanomaterials stimulate immune responses or alter immune pathways to increase radiosensitivity and amplify the effects of immunotherapies. Recent studies demonstrated that nanoparticles can deliver immune checkpoint inhibitors, cytokines, and STING agonists directly into the tumor microenvironment, thereby reprogramming immune responses and amplifying radiosensitization.

Intelligent nanosystems can target resistant cancer cell subpopulations and overcome the biological barriers. Advanced nanocarriers enable precise drug delivery, improved tumor penetration, and reduced systemic toxicity, particularly through ligand-mediated active targeting strategies such as antibodies and aptamers. Polymer-based nanoparticles and liposomes have emerged as research hotspots because of their controlled release properties, multifunctional loading capabilities, and surface modification potential. Inhibitors targeting DNA damage repair mechanisms or tumor hypoxia can further enhance radiotherapy outcomes. The development of stimuli-responsive nanocarriers, which release payloads in response to pH, heat, light, or redox conditions, allows for the precise spatiotemporal control of therapeutic delivery. Moreover, advances in biodegradable materials, scalable synthesis protocols, and immune-evasive coatings (e.g., PEGylation and stealth lipids [57]) are crucial for improving biosafety and translational feasibility. Multifunctional theragnostic nanoparticles capable of simultaneously imaging and treating tumors have become a central research focus in real-time monitoring and personalized therapy.

Overcoming challenges related to scalability and safety is crucial for the clinical translation of these innovations. The key steps include standardizing manufacturing, improving biocompatibility, and conducting large-scale clinical trials. Collaborative research efforts and investments in emerging technologies will facilitate nanotechnology-driven radiotherapy as a safer and more effective cornerstone of cancer treatment.

Overall, the use of nanomaterials as a bridge to combine radiotherapy with other therapies, such as immunotherapy or photothermal therapy, is projected to become a major trend, offering synergistic effects for comprehensive cancer treatment. Although this review focuses on the application of nanomaterials in radiotherapy, future progress will rely on advancements in nanomaterial fabrication technologies, improved biocompatibility, and scalable production processes to support their clinical translation.

### 4.4. Advantages and Limitations

Previous studies predominantly explored the bibliometric aspects of nanomaterials for cancer treatment. This is the first study to comprehensively analyze the intersection between tumor radiotherapy and nanomaterials. However, this study had some limitations. First, it relied solely on the WoSCC database, which may have excluded relevant studies from other sources. Nevertheless, the WoSCC is one of the most extensive and widely recognized databases for bibliometric analysis. Second, only English language publications were included, potentially introducing a selection bias.

## 5. Conclusions

This study used VOSviewer, R, and CiteSpace to analyze the role of nanomaterials in radiotherapy. Our findings highlighted the most influential countries, journals, institutions, and keywords in this field. In addition, we reviewed the current research landscape and future trends. Currently, the application of nanomaterials as radiosensitizers and their integration into comprehensive cancer treatments remain key areas of research. Nanoparticle-based strategies transform radiotherapy by improving tumor specificity, enhancing therapeutic efficacy, and minimizing adverse effects. Although the clinical applications of these strategies are limited, they represent the foundation for more efficient and personalized cancer treatments.

## Figures and Tables

**Table 1:** Bibliometric Summary of the Dataset.

Description	Results
Main Information about Data	
Timespan	2004:2024
Sources (journals, books, etc.)	555
Documents	2022
Annual Growth Rate %	28.16
Document Average Age	3.75
Average Citations per document	36.74
References	110,686
Document Contents	
Keywords Plus (ID)	4176
Author’s Keywords (DE)	4359
Authors	
Authors	14,021
Authors of Single-authored Documents	27
Author Collaboration	
Single-authored documents	28
Co-authors per document	9.97
International Co-authorships %	25.42
Document Types	
article	1333
article; book chapter	21
article; data paper	1
article; early access	25
article; proceedings paper	32
article; retracted publication	2
review	599
review; book chapter	1
review; early access	8

**Table 2:** Top 10 most productive countries/regions from 2004 to 2024.

Rank	Country	Publication (%)	Total Citations	TLS
1	China	1051 (50.41)	47,286	172
2	USA	325 (15.59)	15,404	202
3	India	155 (7.43)	2710	77
4	Australia	66 (3.17)	2993	68
5	Germany	74 (3.55)	2250	62
6	Iran	121 (5.80)	2192	51
7	France	92 (4.41)	4080	45
8	Italy	94 (4.51)	1675	42
9	South Korea	63 (3.02)	1467	34
10	Saudi Arabia	44 (2.11)	1143	31

**Table 3:** Top 10 most productive institutions from 2004 to 2024.

Rank	Institution	Publication	Total Citations	TLS
1	Soochow University	155	14,234	43
2	Chinese Academy of Science	142	11,787	105
3	University of Chinese Academy of Science	65	5172	68
4	Shanghai Jiao Tong University	56	2288	31
5	Fudan University	42	2649	28
6	Nanjing University	41	2450	12
7	Jinan University	41	1692	10
8	Sun Yat-Sen University	35	940	11
9	Nanjing Medical University	32	1333	18
10	University of Science and Technology of China	24	1213	18

**Table 4:** Top 10 most productive authors from 2004 to 2024.

Rank	Authors	Publication	Total Citations	TLS
1	Liu, Zhuang	77	10,861	197
2	Yang, Kai	61	7608	164
3	Yi, Xuan	41	5095	125
4	Liang, Chao	31	5549	120
5	Gu, Zhanjun	29	2646	0
6	Cheng, Liang	25	3970	77
7	Dong, Ziliang	24	3093	86
8	Chao, Yu	23	3249	93
9	Feng, Liangzhu	21	2559	68
10	Song, Guosheng	16	3374	64

**Table 5:** Top 10 most productive journals from 2004 to 2024.

Rank	Journal	Publication	TLS	Total Citation	Average Citation	IF	JCR
1	ACS Nano	128	1145	9255	72.3	15.881	Q1
2	Advanced Materials	29	824	5016	173.0	32.086	Q1
3	Biomaterials	40	476	4075	101.9	15.304	Q1
4	Nano Letters	32	278	2814	87.9	11.189	Q1
5	Advanced Functional Materials	23	274	1852	80.5	19.924	Q1
6	Journal of Materials Chemistry B	33	267	876	26.5	6.331	Q1
7	ACS Applied Materials & Interfaces	32	264	1285	40.2	10.383	Q1
8	Nanoscale	25	260	1171	46.8	7.79	Q1
9	Int. Journal of Nanomedicine	42	258	1439	34.3	6.4	Q1
10	Small	30	248	1513	50.4	13.281	Q1

**Table 6:** Types and Applications of Nanomaterials in Cancer Treatment.

Category	Examples	Key Features/Applications	Stage
Organic-Based Nanocarriers			
Lipid-Based Nanomaterials [46]	Liposomes, SLNs, NLCs	Biocompatible, suitable for hydrophilic and hydrophobic drugs, excellent drug encapsulation capacity	Liposomes: Clinical; Others: Research
Polymer-Based Nanomaterials [47]	Polymeric nanoparticles, micelles, dendrimers	Functionalizable for targeted delivery, controlled release, and multifunctionality	Some PLGA-based: Clinical; Others: Research
Inorganic-Based Nanocarriers			
Metal-Based Nanomaterials [48]	Gold nanoparticles (AuNPs), magnetic nanoparticles (Fe_3_O_4_)	Thermal therapy, magnetic targeting, imaging capabilities	Magnetic nanoparticles: Clinical; Gold nanoparticles: Research
Silica-Based Nanomaterials [49]	Mesoporous silica nanoparticles (MSNs)	High surface area and porous structure, good for loading/release	Research
Carbon-Based Nanomaterials [50]	Graphene, CNTs, fullerenes	Unique mechanical and chemical properties for drug delivery	Research
Biological-Based Nanocarriers			
Protein-Based Nanomaterials [51]	Albumin nanoparticles	Long-term drug delivery, improved drug stability	Clinical (e.g., Abraxane)
Polysaccharide-Based Nanomaterials [52]	Chitosan, hyaluronic acid	Biodegradable, suitable for targeted delivery	Research
Stimuli-Responsive Nanocarriers			
pH-Responsive Nanomaterials [53]	–	Release drugs in specific pH environments (e.g., tumors)	Research
Temperature-Responsive Nanomaterials [54]	Thermosensitive polymers	Release drugs in response to heat	Research
Light/Magnetic/Ultrasound-Responsive [55]	Drug delivery + imaging	Controlled drug release under external stimuli (light, magnetic fields, ultrasound)	Research
Multifunctional Nanocarriers			
Theragnostic Nanoparticles [56]	Drug delivery + imaging	Combines drug delivery and imaging (diagnostic + therapeutic)	Research
Synergistic Treatment [41,57]	Combination of nano-materials with drugs, RNA, proteins, or immunotherapy molecules	Enhances therapeutic efficiency through multifunctionality	Research

## Data Availability

Data are available from the corresponding author upon request.

## Supplementary Materials

The following supporting information can be downloaded at: https://www.mdpi.com/article/10.3390/nano15151205/s1.

## Abbreviations

The following abbreviations are used in this manuscript:

Doxil	Doxorubicin liposome injection
FDA	Food and Drug Administration
GNPs	Gold nanoparticles
WoSCC	Web of Science Core Collection
IFs	Impact factors
JCR	Journal Citation Reports
LSs	Link strengths
TLS	Total link strength
ACS Nano	American Chemical Society Nano
PARP	Poly(ADP-ribose) polymerase
ATM	Ataxia-telangiectasia mutated
ATR	ATM- and Rad3-related
TME	Tumor microenvironment
MRNPs	Metallic radioactive nanoparticles
Au	Gold
Ag	Silver
Pt	Platinum
CNTs	Carbon nanotubes
GO	Graphene oxide
PLGA	Poly(lactic-co-glycolic acid)
PEG	Polyethylene glycol
MRI	Magnetic resonance imaging
SiNPs	Silica-based nanoparticles
