# Supplementary material for: Current Research Trends and Hotspots in Radiotherapy Combined with Nanomaterials for Cancer Treatment: A Bibliometric and Visualization Analysis
Source: Nanomaterials (Basel). 2025 Aug 6;15(15):1205. doi: 10.3390/nano15151205 (PMC12348538; doi:10.3390/nano15151205)
Supplement: Supplementary file 1 [file nanomaterials-15-01205-s001.zip › nanomaterials-3758173-supplementary.pdf]

Table S1： The details of the 64 highest-cited publications.

Rank	Publication year	Authors	Titals	Journals	Total Citation	Types
**1**	2017	Liu, JN	Chemical Design and Synthesis of Functionalized Probes for Imaging and Treating Tumor Hypoxia	CHEMICAL REVIEWS	607	Review
**2**	2015	Pérez-Herrero, E	Advanced targeted therapies in cancer: Drug nanocarriers, the future of chemotherapy	EUROPEAN JOURNAL OF PHARMACEUTICS AND BIOPHARMACEUTICS	562	Review
**3**	2018	Gai, SL	Recent advances in functional nanomaterials for light-triggered cancer therapy	NANO TODAY	505	Review
**4**	2021	An, D	NIR-II Responsive Inorganic 2D Nanomaterials for Cancer Photothermal Therapy: Recent Advances and Future Challenges	ADVANCED FUNCTIONAL MATERIALS	444	Review
**5**	2022	Jia, CY	Chemodynamic Therapy via Fenton and Fenton-Like Nanomaterials: Strategies and Recent Advances	SMALL	420	Review
**6**	2023	Zeng, LL	Advancements in nanoparticle-based treatment approaches for skin cancer therapy	MOLECULAR CANCER	384	Review
**7**	2023	Zhu, YJ	Rational Design of Biomaterials to Potentiate Cancer Thermal Therapy	CHEMICAL REVIEWS	361	Review

**8**	2023	Ashrafizadeh, M	(Nano)platforms in breast cancer therapy: Drug/gene delivery, advanced nanocarriers and immunotherapy	MEDICINAL RESEARCH REVIEWS	343	Review
**9**	2024	Ashrafizadeh, M	Molecular panorama of therapy resistance in prostate cancer: a pre-clinical and bioinformatics analysis for clinical translation	CANCER AND METASTASIS REVIEWS	332	Review
**10**	2023	Zhu, XD	Nanomaterials in tumor immunotherapy: new strategies and challenges	MOLECULAR CANCER	319	Review
**11**	2020	Chen, JM	Advances in nanomaterials for photodynamic therapy applications: Status and challenges	BIOMATERIALS	307	Review
**12**	2020	Ding, BB	Manganese Oxide Nanomaterials: Synthesis, Properties, and Theranostic Applications	ADVANCED MATERIALS	285	Review
**13**	2019	Xie, JN	Emerging Strategies of Nanomaterial-Mediated Tumor Radiosensitization	ADVANCED MATERIALS	285	Review
**14**	2018	Wang, H	Cancer Radiosensitizers	TRENDS IN PHARMACOLOGICAL SCIENCES	282	Review
**15**	2023	Fan, HH	Tumor microenvironment-responsive manganese-based nanomaterials for cancer treatment	COORDINATION CHEMISTRY REVIEWS	275	Review
**16**	2017	Song, GS	Emerging Nanotechnology and Advanced Materials for Cancer Radiation Therapy	ADVANCED MATERIALS	274	Review
**17**	2022	Li, TZ	Glioma diagnosis and therapy: Current challenges and nanomaterial-based solutions	JOURNAL OF CONTROLLED RELEASE	268	Review

**18**	2019	Chen, JQ	Nanomaterials as photothermal therapeutic agents	PROGRESS IN MATERIALS SCIENCE	265	Review
**19**	2023	Li, YT	Advances and challenges in the treatment of lung cancer	BIOMEDICINE & PHARMACOTHERAPY	263	Review
**20**	2019	Lan, MH	Photosensitizers for Photodynamic Therapy	ADVANCED HEALTHCARE MATERIALS	248	Review
**21**	2016	Kemp, JA	Combo nanomedicine: Co-delivery of multi-modal therapeutics for efficient, targeted, and safe cancer therapy	ADVANCED DRUG DELIVERY REVIEWS	245	Review
**22**	2022	Alamdari, SG	Recent advances in nanoparticle-based photothermal therapy for breast cancer	JOURNAL OF CONTROLLED RELEASE	225	Review
**23**	2021	Gong, LY	Application of Radiosensitizers in Cancer Radiotherapy	INTERNATIONAL JOURNAL OF NANOMEDICINE	223	Review
**24**	2024	Nag, S	Nanomaterials-assisted photothermal therapy for breast cancer: State-of-the-art advances and future perspectives	PHOTODIAGNOSIS AND PHOTODYNAMIC THERAPY	221	Article
**25**	2020	Chen, Y	Gold Nanoparticles as Radiosensitizers in Cancer Radiotherapy	INTERNATIONAL JOURNAL OF NANOMEDICINE	217	Review

**26**	2020	Filipczak, N	Recent advancements in liposome technology	ADVANCED DRUG DELIVERY REVIEWS	201	Review
**27**	2020	Cheng, L	2D Nanomaterials for Cancer Theranostic Applications	ADVANCED MATERIALS	197	Review
**28**	2023	Nejabat, M	An Overview on Gold Nanorods as Versatile Nanoparticles in Cancer Therapy	JOURNAL OF CONTROLLED RELEASE	173	Review
**29**	2023	Jiang, ZY	Stimuli responsive nanosonosensitizers for sonodynamic therapy	JOURNAL OF CONTROLLED RELEASE	173	Review
**30**	2023	He, MY	Reactive oxygen species-powered cancer immunotherapy: Current status and challenges	JOURNAL OF CONTROLLED RELEASE	162	Article
**31**	2021	Li, JC	Electromagnetic Nanomedicines for Combinational Cancer Immunotherapy	ANGEWANDTE CHEMIE-INTERNATIONAL EDITION	162	Review
**32**	2023	Duan, SF	Nanomaterials for photothermal cancer therapy	RSC ADVANCES	159	Review
**33**	2018	Chang, D	Biologically Targeted Magnetic Hyperthermia: Potential and Limitations	FRONTIERS IN PHARMACOLOGY	152	Review
**34**	2023	Zhen, WY	Nanoparticle-Mediated Radiotherapy Remodels the Tumor Microenvironment to Enhance Antitumor Efficacy	ADVANCED MATERIALS	151	Review
**35**	2019	Chen, Q	Local biomaterials-assisted cancer immunotherapy to trigger systemic antitumor responses	CHEMICAL SOCIETY REVIEWS	149	Review

**36**	2022	Xu, DT	Catalase-Like Nanozymes: Classification, Catalytic Mechanisms, and Their Applications	SMALL	139	Review
**37**	2023	Yang, J	Multifunctional metal-organic framework (MOF)-based nanoplatforms for cancer therapy: from single to combination therapy	THERANOSTICS	135	Review
**38**	2020	Liu, XL	Comprehensive understanding of magnetic hyperthermia for improving antitumor therapeutic efficacy	THERANOSTICS	135	Review
**39**	2024	Deng, YJ	Prospects, advances and biological applications of MOF-based platform for the treatment of lung cancer	BIOMATERIALS SCIENCE	131	Review
**40**	2022	Ren, XY	Nanozymes-recent development and biomedical applications	JOURNAL OF NANOBIOTECHNOLOGY	126	Review
**41**	2024	Li, X	Pillararene-Based Stimuli-Responsive Supramolecular Delivery Systems for Cancer Therapy	ADVANCED MATERIALS	117	Review
**42**	2023	Chen, XL	Current and promising applications of Hf(iv)-based MOFs in clinical cancer therapy	JOURNAL OF MATERIALS CHEMISTRY B	115	Review
**43**	2022	Muhammad, P	Carbon dots supported single Fe atom nanozyme for drug-resistant glioblastoma therapy by activating autophagy-lysosome pathway	NANO TODAY	80	Article

**44**	2021	Yang, GB	Multifunctional MnO2 nanoparticles for tumor microenvironment modulation and cancer therapy	WILEY INTERDISCIPLINARY REVIEWS-NANOMEDICINE AND NANOBIOTECHNOLOGY	75	Review
**45**	2018	Zhu, P	Nanoenzyme-Augmented Cancer Sonodynamic Therapy by Catalytic Tumor Oxygenation	ACS NANO	71	Article
**46**	2015	Yong, Y	Tungsten Sulfide Quantum Dots as Multifunctional Nanotheranostics for In Vivo Dual-Modal Image-Guided Photothermal/Radiotherapy Synergistic Therapy	ACS NANO	70	Article
**47**	2020	Wang, J	Applications of Inorganic Nanomaterials in Photothermal Therapy Based on Combinational Cancer Treatment	INTERNATIONAL JOURNAL OF NANOMEDICINE	68	Review
**48**	2023	Singh, AV	Artificial intelligence and machine learning disciplines with the potential to improve the nanotoxicology and nanomedicine fields: a comprehensive review	ARCHIVES OF TOXICOLOGY	67	Review
**49**	2021	Huang, ZS	Nanoscale coordination polymers induce immunogenic cell death by amplifying radiation therapy mediated oxidative stress	NATURE COMMUNICATIONS	65	Article

**50**	2019	Chen, Q	Nanoparticle-Enhanced Radiotherapy to Trigger Robust Cancer Immunotherapy	ADVANCED MATERIALS	60	Article
**51**	2016	Song, GS	Perfluorocarbon-Loaded Hollow Bi2Se3 Nanoparticles for Timely Supply of Oxygen under Near-Infrared Light to Enhance the Radiotherapy of Cancer	ADVANCED MATERIALS	59	Article
**52**	2020	Ma, WJ	Coating biomimetic nanoparticles with chimeric antigen receptor T cell-membrane provides high specificity for hepatocellular carcinoma photothermal therapy treatment	THERANOSTICS	55	Article
**53**	2017	Chen, J	Oxygen-Self-Produced Nanoplatform for Relieving Hypoxia and Breaking Resistance to Sonodynamic Treatment of Pancreatic Cancer	ACS NANO	52	Article
**54**	2023	Deng, Z	Biomineralized MnO2 Nanoplatforms Mediated Delivery of Immune Checkpoint Inhibitors with STING Pathway Activation to Potentiate Cancer Radio-Immunotherapy	ACS NANO	50	Article
**55**	2021	Sun, LL	ATP-Responsive Smart Hydrogel Releasing Immune Adjuvant Synchronized with Repeated Chemotherapy or Radiotherapy to Boost Antitumor Immunity	ADVANCED MATERIALS	50	Article

**56**	2016	Song, XJ	Ultrasound Triggered Tumor Oxygenation with Oxygen-Shuttle Nanoperfluorocarbon to Overcome Hypoxia-Associated Resistance in Cancer Therapies	NANO LETTERS	49	Article
**57**	2019	Dong, ZL	Amplification of Tumor Oxidative Stresses with Liposomal Fenton Catalyst and Glutathione Inhibitor for Enhanced Cancer Chemotherapy and Radiotherapy	NANO LETTERS	48	Article
**58**	2021	Wang, C	Maintaining manganese in tumor to activate cGAS-STING pathway evokes a robust abscopal anti-tumor effect	JOURNAL OF CONTROLLED RELEASE	47	Article
**59**	2017	Gao, M	Erythrocyte-Membrane-Enveloped Perfluorocarbon as Nanoscale Artificial Red Blood Cells to Relieve Tumor Hypoxia and Enhance Cancer Radiotherapy	ADVANCED MATERIALS	47	Article
**60**	2016	Liu, JJ	Nanoscale metal-organic frameworks for combined photodynamic & radiation therapy in cancer treatment	BIOMATERIALS	46	Article
**61**	2018	Sun, SK	Engineering Persistent Luminescence Nanoparticles for Biological Applications: From Biosensing/Bioimaging to Theranostics	ACCOUNTS OF CHEMICAL RESEARCH	43	Review
**62**	2016	Song, GS	Catalase-Loaded TaOx Nanoshells as Bio-Nanoreactors Combining High-Z Element and Enzyme Delivery for Enhancing Radiotherapy	ADVANCED MATERIALS	41	Article

**63**	2023	Shen, WH	A Polymeric Hydrogel to Eliminate Programmed Death-Ligand 1 for Enhanced Tumor Radio-Immunotherapy	ACS NANO	40	Article
**64**	2018	Liu, YY	Modulating Hypoxia via Nanomaterials Chemistry for Efficient Treatment of Solid Tumors	ACCOUNTS OF CHEMICAL RESEARCH	37	Review
